# Stimulating the Facial Nerve to Treat Ischemic Stroke: A Systematic Review

**DOI:** 10.3389/fneur.2021.753182

**Published:** 2021-11-18

**Authors:** Turner S. Baker, Justin Robeny, Danna Cruz, Alexis Bruhat, Alfred-Marc Iloreta, Anthony Costa, Thomas James Oxley

**Affiliations:** ^1^Department of Neurosurgery, Icahn School of Medicine at Mount Sinai, New York, NY, United States; ^2^Sinai BioDesign, Icahn School of Medicine at Mount Sinai, New York, NY, United States; ^3^The Grove School of Engineering, The City College of New York, New York, NY, United States; ^4^Department of Otolaryngology, Icahn School of Medicine at Mount Sinai, New York, NY, United States

**Keywords:** animal studies, cerebral blood flow, cerebrovascular disease, vascular surgery, stroke

## Abstract

Acute ischemic stroke (AIS) is a common devastating disease that has increased yearly in absolute number of cases since 1990. While mechanical thrombectomy and tissue plasminogen activator (tPA) have proven to be effective treatments, their window-of-efficacy time is very short, leaving many patients with no viable treatment option. Over recent years there has been a growing interest in stimulating the facial nerves or ganglions to treat AIS. Pre-clinical studies have consistently demonstrated an increase in collateral blood flow (CBF) following ganglion stimulation, with positive indications in infarct size and neurological scores. Extensive human trials have focused on trans-oral electrical stimulation of the sphenopalatine ganglion, but have suffered from operational limitations and non-significant clinical findings. Regardless, the potential of ganglion stimulation to treat AIS or elongate the window-of-efficacy for current stroke treatments remains extremely promising. This review aims to summarize results from recent trial publications, highlight current innovations, and discuss future directions for the field. Importantly, this review comes after the release of four important clinical trials that were published in mid 2019.

## Introduction

### Acute Ischemic Stroke

Stroke is the leading cause of disability and the fifth leading cause of death in the United States (US). Approximately 795,000 people experience a new or recurrent stroke each year (1). Acute ischemic stroke (AIS) occurs when an obstruction within a blood vessel decreases cerebral blood flow, depriving nerve cells of oxygen and leading to severe metabolic failure and neural death (2–4). Immediately following stroke, a section of the brain referred to as the ischemic core is subject to extreme hypoxia, leading to irreversible brain damage (5). The area surrounding the ischemic core, the ischemic penumbra, is severely hypoperfused and non-functioning, yet can regain functionality if blood flow is restored to the area (5, 6). This recovery is highly time-dependent, as the penumbra rapidly evolves into the ischemic core (6, 7). The recovery of the penumbra has been demonstrated to have a significant effect on clinical outcomes; Meretoja et al. showed that for every 20-min reduction in time to reperfusion increases the average disability-free life span by 3 months (8).

### Current Treatments: Endovascular Thrombectomy and Tissue Plasminogen Activator

Management for AIS relies on rapid treatment times to avoid penumbra evolution. The goal of modern stroke treatment facilities is to reperfuse the ischemic area via endovascular thrombectomy, mechanically removing the blood clot with catheter-based devices (9). The faster the patient achieves reperfusion, the more likely the patient will have excellent neurological outcomes at 90 days (10). The second treatment paradigm for AIS is the use of intravenous (IV) tissue plasminogen activator (tPA), which acts to chemically break down clots (11–14). IV-tPA is commonly used prior to patient transport for thrombectomy.

Both endovascular thrombectomy and IV-tPA suffer from a limited window-of-efficacy. Currently, stroke guidelines list the acceptable window of treatment for mechanical thrombectomy at 24 h (15, 16), and a recommended IV-tPA door-to-treatment time of 60 min (10, 17, 18). These windows are frequently missed, with fewer than a third of patients in the U.S. treated within the IV-tPA 60 min window (19). Mandatory neuroimaging, presence of a highly-trained neurointerventionalist, and hospital transfer times all reduce event-to-treatment times and result in reductions of successful functional outcomes following recanalization (16, 20, 21).

Thrombectomy is a well-established treatment (15, 16, 22), but patients routinely fail to achieve functional independence (mRS ≤ 2) due to extensive event-to-treatment times (15, 16). There is a clear need for innovative approaches to extend the window-of-efficacy for endovascular thrombectomy and IV-tPA.

### Time Is Brain: Inhibiting the Evolution of the Ischemic Penumbra

Researchers have recently sought to find new approaches to arrest the evolution of the ischemic penumbra and keep this susceptible region from becoming irreversibly damaged. Current approaches aim to either enhance oxygen delivery to the penumbra or reduce tissue oxygen demand (6). In addition to door-to-treatment time limitations, many patients also become ineligible for mechanical thrombectomy due to large ischemic core volumes. Inhibiting the evolution of the penumbra may help buy time by limiting core volume growth from reaching recommended exclusionary levels (23, 24). Inhibition of penumbra evolution could also be highly beneficial when combined with IV-tPA, potentially increasing its effectiveness and elongating its window-of-efficacy (6).

#### Increased Collateral Blood Circulation Through Ganglion Stimulation

**Figure 1:**
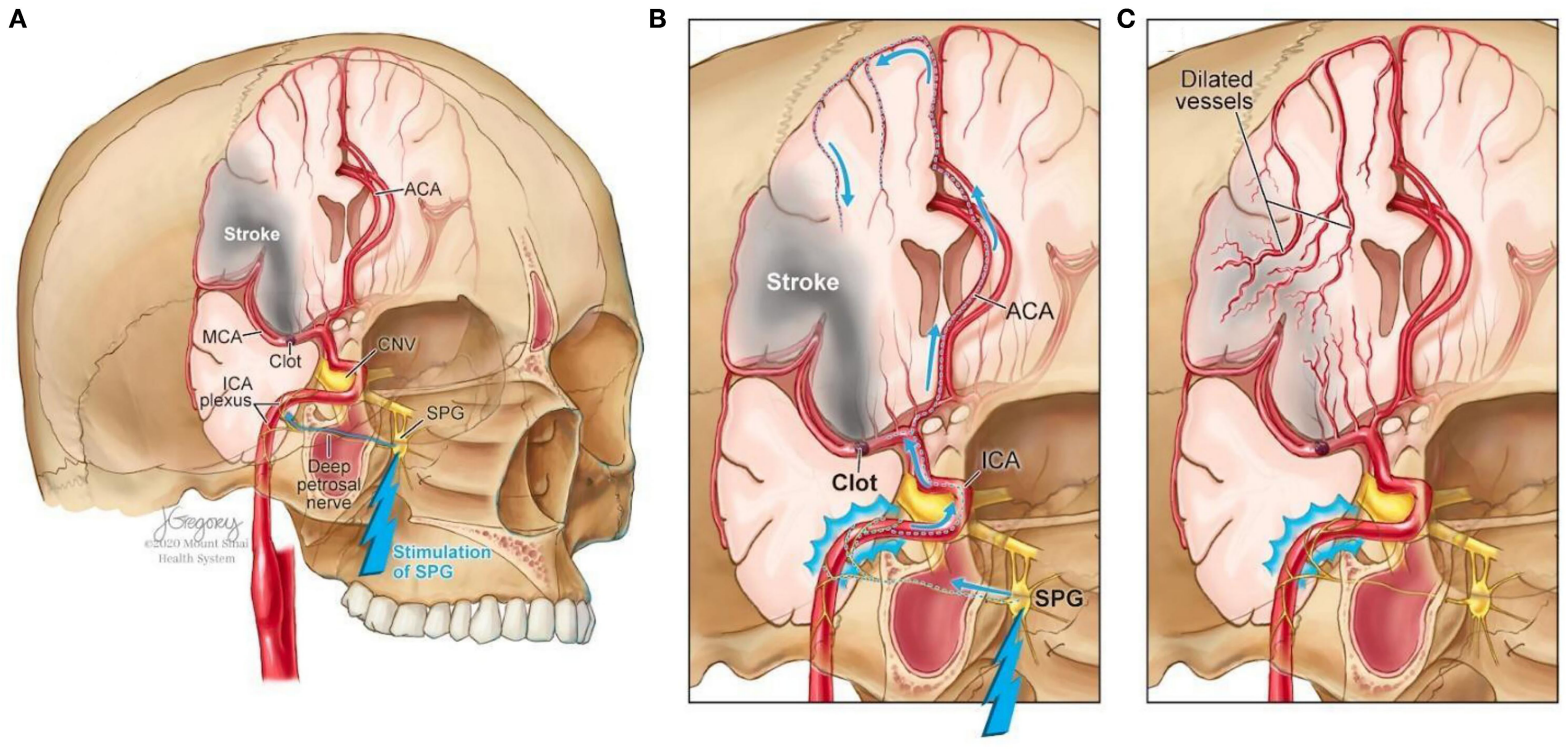
Increased collateral blood circulation by sphenopalatine ganglion (SPG) stimulation. **(A)** Anatomical overview of stroke affected brain region before SPG stimulation. **(B)** The SPG contains parasympathetic fibers that synapse in the ganglion and innervate the internal carotid artery (ICA) through the deep petrosal nerve. **(C)** SPG stimulation induces vasodilation of blood vessels in the anterior circulation, increasing blood flow to the affected area limiting the evolution of the penumbra and reducing infarct volume.

Facial nerve-induced vasodilation of the cerebral arteries is an emerging therapeutic target that seeks to increase collateral blood flow in ischemic brain tissue, improve oxygen availability, and improve patient functional outcomes after stroke. Collateral circulation refers to alternative, pre-existing vascular pathways that deliver blood to target tissue when the primary vessel is occluded (25). Imaging of the brain and vessels has shown that collateral blood flow can preserve brain tissue for hours after major arteries to the brain are blocked (26). Figure 1 illustrates how facial nerve-induced increased collateral blood flow has the ability to ameliorate clot-induced tissue death by limiting ischemic penumbra evolution.

The sphenopalatine ganglion (SPG), also known as the pterygopalatine ganglion, is the largest and most superior ganglion of the sympathetic and parasympathetic nervous system and contains the largest collection of neurons in the calvarium outside of the brain (27). Humans have two SPGs, on each side of the midface, located within the viscerocranium in a space called the pterygopalatine fossa. This fossa has direct connections to the middle cranial fossa, nasal cavity, orbit, infratemporal fossa and oral cavity (28, 29). The SPG contains parasympathetic fibers that synapse in the ganglion and innervate the internal carotid artery (ICA) through the deep petrosal nerve (30). Electrical stimulation of the SPG activates the fibers, releasing several neurotransmitters such as acetylcholine, vasoactive intestinal polypeptide, peptide histidine isoleucine, and nitrous oxide that play a role in inducing vasodilation of blood vessels in the anterior circulation (26, 31).

The geniculate ganglion is a small collection of somatosensory and gustatory ganglion cells (32) located within the temporal bone along the axis of the ear canal. Similar to the SPG, the geniculate ganglion has parasympathetic connections to cerebral arteries and has also been demonstrated to increase cerebral blood flow (33, 34). Its curved shape allows for greater susceptibility to be activated location makes it easier to access through non-invasive routes (33, 35).

## Methods

### Search Strategy and Selection Criteria

The literature search was conducted in three electronic databases MEDLINE, EMBASE, and SCOPUS, to obtain pre-clinical and clinical studies dating from 1986 to 2019. Search terms included “isch(a)emic,” “brain,” “cerebr^*^,” “core,” and “penumbra” with intervention approach terms such as “parasympathetic,” “ganglion,” “facial nerve,” “stimulation,” “neuromodulate^*^,” “electrical acupuncture,” and “magnet^*^.”

Studies selected for final review included those assessing the effects of stimulation in the ischemic stroke indication, as well as the effects on blood flow in the brain from ganglion stimulation. The inclusion criteria for pre-clinical studies had at least one of the following outcome measures: (1) healthy and ischemic stroke-induced animal models, (2) invasive or non-invasive ganglia stimulation, and/or (3) studies providing at least one outcome measurement of cerebral or collateral blood flow, brain infarct size, neuronal survival, and neurological outcomes. Respectively, the inclusion criteria for clinical studies had at least one of the following outcome measures: (1) healthy human volunteers or patients with ischemic stroke, (2) invasive or non-invasive ganglia stimulation, (3) sham stimulation control group, (4) safety and tolerability of intervention, and/or (5) studies providing at least one outcome measurement of cerebral or collateral blood flow, brain infarct size, neuronal survival, functional and neurological outcomes including but not limited to mRS at 90 days post-stroke.

Pre-clinical and clinical studies were excluded if they evaluated facial nerve stimulation for other indications such as hemorrhagic stroke, chronic headache, migraine, rhinitis, or stroke rehabilitation. Reviews, meta-analyses, observational studies, and commentaries were also excluded.

### Risk of Bias Analysis

The final emerging pre-clinical studies were subjected to the Systematic Review Center for Laboratory Animal Experimentation (SYRCLE) risk of bias tool (36). The SYRCLE's tool for assessing risk of bias contains 10 domains which fall under six types of bias; selection bias, performance bias, detection bias, attrition bias, reporting bias, and other. Judgement of bias was indicated as low by a positive (“yes”), high by a negative (“no”), and imprecise (“unclear”) when details reported were insufficient. Physiotherapy Evidence Database (PEDro) scale was selected as the risk of bias assessment tool for clinical studies (37). The PEDro scale consists of 10 items; they are random allocation, concealment of allocation, baseline equivalence, blind subjects, blind therapists, blind assessors, intention to treat analysis, adequate follow-up, between-group statistical analysis, and measurement of data variability and point estimates. Studies with a PEDro score of more than 6 were considered good quality.

## Results

**Figure 2:**
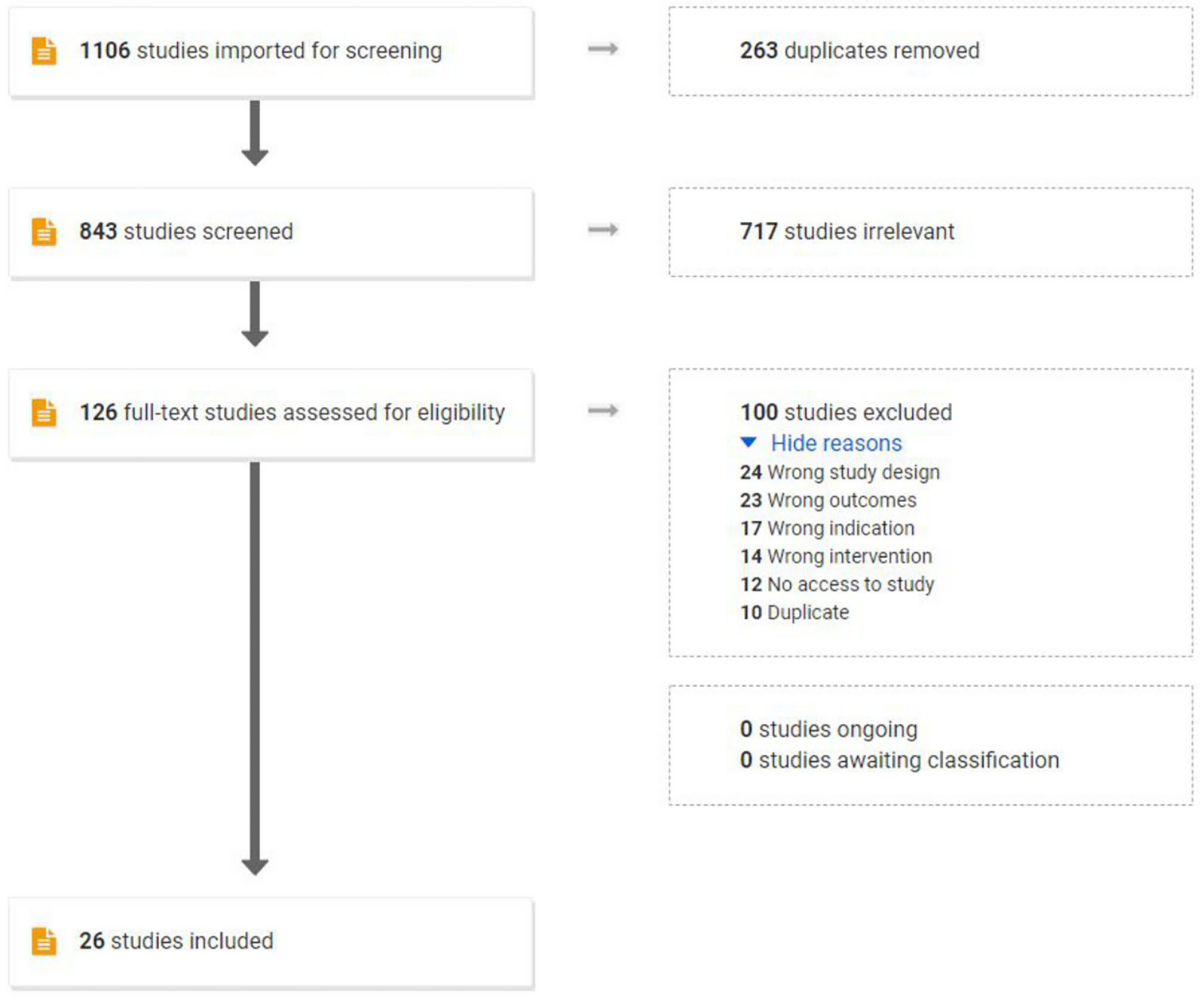
Flow chart of study selection using PRISMA guidelines.

The initial search yielded 1,106 studies: 263 duplicates were removed, 843 studies were screened by title and abstract, and 717 studies were deemed irrelevant articles. A total of 126 studies were assessed for eligibility and were subjected to the predefined inclusion and exclusion criteria via full-text review, resulting in the removal of 100 studies. The final emerging 26 studies combined in this review assessed 22 pre-clinical studies and 5 human studies. One publication contained both a human and a pre-clinical trial. Figure 2 outlines the flow of study selection.

### Quality of Studies

The methodological quality assessments of included pre-clinical and clinical studies are provided in Tables 1, 2, respectively. Animal studies assessed by the SYRCLE found a prevalence of 41.3% for items classified as “unclear,” and 5.9% for items classified as “no” (Table 1). Human trials assessed by the PEDro scale had an average score of 6.0 with scores ranging from 4 to 7 (Table 2).

**Table 1:** Quality assessment of included pre-clinical studies using SYRCLE's tool.

**Study**	**Selection bias**		**Performance bias**			**Detection bias**		**Attrition bias**	**Reporting bias**	**Other**
	**Sequence generation**	**Baseline characteristics**	**Allocation concealment**	**Random housing**	**Blinding**	**Random outcome assessment**	**Blinding**	**Incomplete outcome data**	**Selective outcome reporting**	**Other sources of bias**
Ay et al. (38)	Yes	Yes	Yes	Unclear	No	Yes	Yes	Yes	Yes	Yes
Ay et al. (39)	Yes	Yes	Yes	Unclear	No	Yes	Yes	Yes	Yes	Yes
Ay and AY (40)	Unclear	Yes	Unclear	Unclear	No	Yes	Yes	Yes	Yes	Yes
Ayajiki et al. (41)	Unclear	Yes	Unclear	Unclear	Yes	Yes	Unclear	Yes	Unclear	Yes
Bar-Shir et al. (42)	Unclear	Yes	Unclear	Unclear	Unclear	Yes	Yes	Yes	Yes	Yes
Borsody et al. (43)	Yes	Yes	Yes	Unclear	Yes	Yes	No	Yes	Unclear	Yes
Borsody et al. (44)	Yes	Yes	Yes	Unclear	Yes	Yes	No	Yes	Unclear	Yes
Chi et al. (45)	Yes	Yes	Yes	Unclear	Yes	Yes	Yes	Yes	Yes	Yes
D'Alecy and Rose (46)	Unclear	Yes	Unclear	Unclear	No	Unclear	No	Yes	Unclear	Yes
Edvinsson et al. (47)	Unclear	Yes	Unclear	Unclear	Unclear	Unclear	Unclear	Unclear	Unclear	Yes
Goadsby et al. (48)	No	Unclear	No	Unclear	Unclear	Unclear	Unclear	Unclear	Unclear	Yes
Goadsby et al. (49)	Unclear	Yes	Unclear	Unclear	Unclear	Unclear	Unclear	Unclear	Unclear	Yes
Goadsby et al. (50)	Unclear	Yes	Unclear	Unclear	Unclear	Unclear	Unclear	Unclear	Unclear	Yes
Gulturk et al. (51)	Unclear	Yes	Unclear	Unclear	Yes	Yes	Yes	Yes	Yes	Yes
Gürelik et al. (52)	Unclear	Yes	Unclear	Unclear	Yes	Yes	Yes	Yes	Yes	Yes
Henninger (53)	Unclear	Yes	Unclear	Unclear	Yes	Unclear	Yes	Yes	Yes	Yes
Kuo (54)	Unclear	Yes	Unclear	Unclear	Unclear	Unclear	Unclear	Unclear	Unclear	Yes
Levi et al. (55)	Yes	Yes	Yes	Unclear	No	Yes	Unclear	Unclear	Unclear	Yes
Sanchez et al. (56)	Yes	Yes	Yes	Yes	Yes	Yes	Yes	Yes	Yes	Yes
Seylaz et al. (57)	No	Yes	No	Unclear	No	Unclear	Unclear	Yes	Unclear	Yes
Suzuki et al. (58)	Unclear	Yes	Unclear	Unclear	Unclear	Yes	Yes	Yes	Unclear	Yes
Talman (59)	Unclear	Yes	Unclear	Unclear	Yes	Unclear	Unclear	Yes	Yes	Yes

**Table 2:** Quality assessment of included clinical studies using PEDro.

**Study**	**Random allocation**	**Concealed allocation**	**Baseline comparability**	**Blind subjects**	**Blind therapists**	**Blind assessors**	**Adequate follow-up**	**Intention-to-treat analysis**	**Between group comparisons**	**Point estimates and variability**	**Total Scores**
Khurana et al. (60) (ImpACT-1)	0	0	1	0	0	0	1	1	1	1	5/10
Bornstein 2019 (ImpACT-24A) (61)	1	0	1	0	1	1	1	0	1	1	7/10
Bornstein 2019 (ImpACT-24B) (62)	1	0	1	0	1	1	1	0	1	1	7/10
Saver et al. (ImpACT-24M) (63)	0	0	1	0	0	0	1	1	1	1	5/10
Sanchez et al. (52)	0	0	0	0	0	0	1	1	1	1	4/10

### Invasive Ganglion Stimulation: Pre-clinical Studies

Of the 22 pre-clinical studies included in this review, a majority (38–43, 46–50, 53–55, 57–59) were invasive electrical stimulation procedures (Table 3). Targeted ganglions included SPG, geniculate ganglion (43, 44, 56), trigeminal ganglion (48–52), vagus nerve (38–40, 45), petrosal nerve (46), nasociliary nerve (47), and dorsal facial area (54).

**Table 3:** Characteristics of invasive animal studies.

**References**	**Experimental model**	**Intervention**	**Protocol of stimulation**	**Ganglion target**	**Outcomes of interest**	**Results**
Ay et al. (38)	Ischemic stroke Rats (*n* = 24)	Invasive electrical stimulation	Frequency: 20 Hz Intensity: 0.5 mA Pulse: 0.5 ms for 30 s Parameter: experimental group 1—every 30 min for 3 h experimental group 2—every 5 min for 1 h	Vagus nerve	Infarct volume Neurological score	Infarct volume: The relative percentage of contralateral hemispheric volume that underwent infarction was 16.2 ± 3.2% in the VNS and 33.0 ± 5.0% in the control arms in experimental group 1 (p < 0.05). The respective values for experimental group 2 were 19.8 ± 0.5% and 37.9 ± 2.6% (*p* < 0.05). Neurological score: The functional score improved by 50% in experimental group 1 and 44% in experimental group 2 (*p* < 0.05 for both groups)
Ay et al. (39)	Ischemic stroke Rats (*n* = 32)	Invasive electrical stimulation	Frequency: 20 Hz Intensity: 0.5 mA Pulse: 0.5 ms for 30 s Parameter: every 5 min for 1 h	Right cervical vagus nerve Left cervical vagus nerve	Infarct volume Neurological score CBF	Infarct volume: Infarct size measurement revealed that the volume of ischemic damage was 41–45% smaller in animals receiving stimulation as compared with control animals. Neurological score: The effect of VNS on tissue outcome was associated with better neurological outcome at both 1- and 3-day time points after the induction of ischemia with a significant difference after 24 h after CBF: Both the right and left VNS caused subtle reduction in CBF during each 30-s stimulation period that quickly returned back to the baseline level at the end of each stimulation cycle.
Ay and Ay (40)	Stroke Rats (*n* = 12)	Invasive electrical stimulation	Frequency: 20 Hz Intensity: 0.5 mA Pulse: 0.5 ms for 30 s Parameter: 5 min intervals for 1 h	Vagus nerve	Infarct volume Neurological score CBF	Infarct volume: VNS reduces infarct volume by ~50% as compared to sham stimulation Neurological score: At 24 h the median neurological scores and IQR in the sham SPGi were 3.0 ± 0.0. VNS treatment were 2.5 ± 0.5 in the txSPGi animals (*p* > 0.05) CBF: The mean reduction in rCBF was marginally responsive to SPG ablation, measuring 33.12 ± 8.06% baseline (*n* = 78) in the txSPGi animals
Ayajiki et al. (41)	Healthy Rats (*n* = 8)	Invasive electrical stimulation	Frequency: 5, 10, 20 Hz Intensity: 10 V Pulse: 1 ms Parameter: 30 s every 3-5 min	Sphenopalatine ganglion	CBF	CBF: nerve stimulation induced a marked increase in CBF together with MABP in a frequency-dependent manner at 5, 10, and 20 hz. The highest significant change of 15.4% ± 4.9 occured in the left parietal cortex (*p* = 0.05)
Bar-Shir et al. (42)	Stroke Rats (*n* = 13)	Invasive electrical stimulation	Frequency: 10 Hz Intensity: 2 mA Pulse: 0.5 ms Parameter: two 60-s-long pulses separated by 12 s off-time, applied every 15 min (8 pulses per hour) for 3 h, for seven consecutive days	Sphenopalatine ganglion	Infarct volume Neurological score	Infarct volume: 21.1 ± 3.5% and 20.1 ± 4.5% for the controls and SPG-stimulated group, respectively (*P* = 0.88). Twenty-eight days post-occlusion, the LV of the treated rats decreased to 12.9 ± 3.1% (*P* = 0.05) while the LV of the controls decreased only to 15.6 ± 3.4% (*P* = 0.05). Despite that the difference between the LVvalues of the two groups at day 28 post-MCAO wasnot statistically significant (*P* = 0.57) Neurological score: Signficant increase from baseline after 8-day period with a difference of 5.6 ± 0.8 for the control rats and 3.8 ± 0.4 for the SPG-treated animals, (*P* = 0.04) after t-MCAO. This difference did not reach statistical significant level at 28 day
Borsody (43)	healthy Sheep (*n* = 6), dogs (*n* = 5)	Invasive electrical stimulation	Frequency: 5, 10, 20 Hz Intensity: 0.5, 2.0, 5.0 mA Pulse: 0.10 ms Parameter: 5 min stimulation duration with 30 min recovery	Geniculate ganglion	CBF	CBF: CBF decreased to a level ~15% below pre-stimulation baseline
D'Alecy and Rose (46)	Healthy Dogs (*n* = 3)	Invasive electrical stimulation	Frequency: 2, 5, 10, 20, 40 Hz Intensity: Maximized at 20 Hz Pulse: 3 ms Parameter: 90 s duration	Petrosal nerve	CBF	CBF: Stimulation of the major petrosal nerve produced a frequency-dependent increase in cerebral blood flow that reached a maximum of approximately an 11 % increase in flow
Edvinsson et al. (47)	Healthy Cats (*n* = 6)	Invasive electrical stimulation	Frequency: 0.5, 1.5, 10, 20 Hz Intensity: 100 μA Pulse: 250 μs Parameter:	Nasociliary nerve	CBF	CBF: Stimulation of the nasociliary nerve resulted in a frequency-dependent increase in CBF. A response could be seen across all frequencies with the maximal effect being at 20 Hz with a 30 ± 6% increase in flow
Goadsby et al. (48)	Healthy Monkey (*n* = 9)	Invasive electrical stimulation	Frequency: 0.2–200 Hz Intensity: 500 μA Pulse: 250, 500 μs Parameter: 15 s stimularion duration	Trigeminal ganglion	CBF	CBF: no effect on bulk flow and resistance in the internal carotid circulation
Goadsby and Hoskin (49)	Healthy Cats (*n* = 6)	Invasive electrical stimulation	Frequency: 5 hz Intensity: 500 uA Pulse: 250 uS Parameter:	Trigeminal ganglion	CBF	CBF: Stimulation of the VIIth nerve led to a marked increase in CBF v (47 ± 7% at 5/s) delta CBF was ~150% change at 200 s of stimulation
Goadsby et al. (50)	Healthy Cats (*n* = 6)	Invasive electrical stimulation	Frequency: 0.5, 1, 2, 5, 10, 20 and 30 Hz Intensity: 500 uA Pulse: 250 uS Parameter: 30s stimulation duration at each frequency	Trigeminal ganglion	CBF	CBF: The mean maximal reduction in resistance was 39 Ž. “5% at 20 rs for the carotid bed and 37” 6% at 20 rs for the cerebral circulation
Henninger et al. (53)	Healthy and Ischemic stroke Rats (*n* = 9)	Invasive electrical stimulation	Frequency: 10 Hz Intensity: 1.9–2.2 mA Pulse: 0.2 ms Parameter: 4 sets of 60 s stimulations separated by 12 s intervals	Sphenopalatine ganglion	Infarct volume Neurological score CBF	Infarct volume: TTC-derived lesion volumes were significantly smaller in stimulated vs. non-stimulated animals (120.4 ± 74.1 mm^3^ vs. 239.3 ± 68.5 mm^3^, respectively). CBF-derived lesion volumes in stimulated animals were ~10% smaller (non-significant, *P* > 0.05) than in non-stimulated controls Neurological score: The 24-h neurological scores (mean ± SD min, max, range) were improved in the SPG group (2.5 ± 0.8, 2, 4.2) relative to controls (3.7 ± 0.8, 3, 5, 2). CBF: In the non-ischemic brain, SPG stimulation significantly elevated CBF predominantly within areas supplied by the anterior cerebral artery (by 0.64 mL/g/min relative to baseline. In the ischemic brain, CBF only marginally increased within the penumbra and core (by up to 0.08 and 0.15 mL/g/min relative to pre-stimulation, respectively)
Kuo et al. (54)	Healthy Cats (*n* = 20)	Invasive electrical stimulation	Frequency: 20 Hz Intensity: 2 V Pulse: 0.5 ms Parameter: 5 s train of rectangular pulses	Dorsal facial area	CBF	CBF: Electrical stimulation of the DFA appeared to increase the regional blood flow of both cerebral hemispheres (intracranial tissues) and to increase predominantly the regional blood flow of extracranial tissues on the side ipsilateral to stimulation
Levi et al. (55)	Ischemic stroke Rats *(n* = 14)	Invasive electrical stimulation	Frequency: 10 Hz Intensity: 1, 2 mA Pulse: 200 uS, 500 uS Parameter: two sets of 60-s stimuli separated by a 12-s interval, followed by 13.6 min of “OFF” time, giving a total treatment time of 180 min	Sphenopalatine ganglion	Infarct size CBF	Infarct size: In SPG-stimulated rats (*n* = 6), both the size of the stained cortical region and the intensity of the dye were smaller compared to those in RB-treated non-stimulated animals (*n* = 6). Rats stimulated either at 15 min or 24 h after photothrombosis showed a significant reduction in loss of brain tissue compared to non-treated controls (37.1 ± 6.0; 20.6 ± 4.8 and 17.9 ± 5.1% reduction in cortical volume compared to the contralateral hemisphere for RB, *n* = 9; RB-SPG-15 min, *n* = 8 and RB-SPG-24 h, *n* = 6, respectively. CBF: Stimulation at 1 mA (200 ms) did not induce a significant increase in vascular diameter or rCBF (*n* = 7), however stimulation at 2 mA resulted in a significant increase in both vascular diameter and rCBF in 12 of 14 (86%) rats. Prolonging the pulse duration to 500 ms was associated with an additional significant increase in diameter and rCBF (Figure 1C). No significant changes in vascular diameter or rCBF were observed in non-stimulated animals during 3 h of recordings (*n* = 6).
Seylaz et al. (57)	Healthy Rats (*n* = 6)	Invasive electrical stimulation	Frequency: 25–50 Hz Intensity: 100–200 uA Pulse: 1 ms Parameter: train duration, 1 s on, 1 s off	Sphenopalatine ganglion	CBF	CBF: The stimulation of the sphenopalatine ganglion provoked an increase in CBF in the ipsilateral parietal cortex by ~50%
Suzuki et al. (58)	Healthy Rats (*n* = 31)	Invasive electrical stimulation	Frequency: 3, 10, 30, 60 Hz Intensity: 5 V Pulse: 0.5 ms Parameter: continuous 90 s stimulation	Sphenopalatine ganglion	CBF	CBF: Stimulation at 10 Hz induced a marked increase of the cortical blood flow (CoBF) on the ipsilateral side, whereas no change was observed on the contralateral side. It reached a maximum mean value of 42.5% at 46 s, and then slightly declined during the remaining stimulation period. Electrical stimulation of the postganglionic fibers at different frequencies revealed a maximal increase in the CoBF at 30 Hz in the control situation (47.2%), but at 10 Hz after scopolamine administration (51.6%)
Talman (59)	Healthy Rats (*n* = 12)	Invasive electrical stimulation	Frequency: 4 Hz Intensity: 2–20 V Pulse: 2 ms Parameter: 80 s with a continuous current	Sphenopalatine ganglion	CBF	CBF: At a maximal stimulus (4 V for 80 s) CBF increased 40.4% from a basal value of 27.7 ± 2.7 LDU to a maximum of 38.9 ± 4.3 LDU (pb0.03) during the stimulus

*CBF, cerebral blood flow; CoBF, cortical blood flow; DFA, dorsal facial area; LDU, laser doppler units; LV, lesion volume; MABP, mean arterial blood pressure; MCAO, middle cerebral artery occluded; mNSS modified neurological severity score; rCBF, regional cerebral blood flow; SPG, sphenopalatine ganglion; TTC, 2,3,5-triphenyltetrazolium chloride; txSPGi, treatment Sphenopalatine-intact; VNS, vagus nerve stimulation*.

Key parameters of stimulation protocols included intensity, frequency, pulse, and duration (Tables 3, 4). Several studies sought to find a dose-response relationship or a maximum tolerated frequency and intensity, causing variability between reported stimulation protocols. Electrical stimulations reported intensity in Amps or Volts, ranging from 0.5 to 5mA and 2 to 20V. CBF significantly increased along with blood pressure in a stimulation frequency-dependent manner (41, 46). Even in studies where this dependency was not clear, lower powers and durations were markedly less effective in increasing CBF (56).

**Table 4:** Characteristics of non-invasive animal studies.

**References**	**Experimental model**	**Intervention**	**Protocol of stimulation**	**Ganglion target**	**Outcomes of interest**	**Results**
Borsody et al. (43)	Healthy Sheep (*n* = 6), Dogs (*n* = 5)	Non-invasive magnetic stimulation	Frequency: 5, 10, 20 Hz Intensity: 0.5, 1.0, 1.5 T Pulse: 280 μs Parameter: 5 min stimulation duration with 30 min recovery	Geniculate ganglion	CBF	CBF: CBF was maximized at 120% baseline CBF at 10 Hz frequency and 1.5 T
Borsody et al. (44)	Stroke Dogs (*n* = 12)	Non-invasive magnetic stimulation	Frequency: 10 Hz Intensity: 1.8 T Pulse: 280 μs Parameter: 5 min stimulation duration with 30 min recovery	Geniculate ganglion	Infarct volume CBF	Infarct volume: The size of ischemic core was statistically smaller in the stimulation group in comparison to the control group (*P* < 0.01) that showed an enlargement of ischemic core volume over time CBF: Average CBF was decreased to ≈70% of baseline levels in the ischemic hemisphere region of interest, and perfusion stayed at those depressed levels in the control group, whereas it was returned to normal by facial nerve stimulation (*P* < 0.01)
Chi et al. (45)	Stroke Rats (*n* = 24)	Non-invasive electrical stimulation	Frequency: 2/15 Hz Intensity: 1.0 mA Pulse: N/A Parameter:30 min stimulation duration	Vagus nerve	Infarct volume Neurological score CBF	Infarct volume: ratio of hemispheric infarct was significantly lowered by EA (13.60 ± 2.20%, *P* < 0.05), and there was no significant difference in the NEA (35.48 ± 3.23%) Neurological score: Lower neurological scores were observed in the EA group as compared to the NEA group CBF: EA induced a constant and stable increase in the CBF to the ischemic area, with a significant difference compared with the other two groups at 20, 25, 30 min (*P* < 0.05).
Gulturk et al. (51)	Healthy Rabbits (*n* = 22)	Non-invasive electrical stimulation	Frequency: 10 Hz Intensity: 5 V Pulse: 0.5 ms Parameter: 90 continuous stimulation	Trigeminal ganglion	CBF	CBF: The maximum increase in right and left CCoBF was 15.6% and 15.1% respectively. The CCoBF values of right hemisphere group were comparable to that of the left hemisphere group.
Gürelik et al. (52)	Healthy Rabbits (*n* = 40)	Non-invasive electrical stimulation	Frequency: 10 Hz Intensity: 5 V Pulse: 0.5 ms Parameter: 90 continuous stimulation	Trigeminal ganglion	CBF	CBF: In experiment group, CBF increased together with the beginning of electrical stimulation. The flow values were remained high as long as the stimulation. Treatment group had 15% increase in CBF as compared to sham and difference was statistically significant.
Sanchez et al. (56)	Healthy Pigs (*n* = 24)	Non-invasive magnetic stimulation	Frequency: 10 Hz Intensity: 1.3 T, 1.6 T, 1.9 T Pulse: 280 μs Parameter: 2, 3.5, 5 min stimulation	Geniculate ganglion	CBF	CBF: The increase in CBF occurred throughout the brain without obvious preference for the hemisphere ipsilateral to stimulation. With stimulation powers ≥ 1.3 Tesla power and durations ≥ 2 min, CBF increased in the range of 30–90% above the pre-stimulation baseline in most stimulation trials. On average, the CBF increased by 77% over baseline.

*CBF, cerebral blood flow; CCoBF, cerebral cortical blood flow; EA, electroacupuncture; MABP, mean arterial blood pressure; NEA, no electroacupuncture*.

Intervention groups undergoing stimulation routinely demonstrated increased CBF. Three studies did not report an increase in CBF, with one not recording CBF (42), one reporting no change (64), and another reporting a decrease following vagus nerve stimulation (39). Goadsby et al. (49) reported the largest shift from baseline following stimulation, reporting a 150% increase following 200 s of stimulation. CBF increased throughout the brain without an obvious preference for the hemisphere ipsilateral to stimulation (54, 56). The ability for stimulation to increase CBF flow was found to be lower in ischemic models compared to CBF increase in health models (39, 40, 53). Animals that underwent invasive stimulation were found to have improved neurological scores in five of the seventeen studies (38, 39, 42, 53, 59). Only one study tracking neurological scores found a decrease following ganglion stimulation, however, they also reported a reduction in infarct volume compared to controls (40). In all studies that reported infarct volume stimulated models performed better (38–40, 42, 53, 55, 59). Bar-Shir et al. (42) found both improved neurological scores and infarct volumes in stimulated models at 8 days compared to control, but no difference in a 28 day follow-up.

### Invasive Ganglion Stimulation: Clinical Studies

Four clinical studies using invasive electrical stimulation to treat AIS were identified (Table 5) (60–63). All four targeted the SPG using an implanted device (ISS, BrainsGate, Israel). The procedure involved surgically placing a platinum-iridium stimulator trans-orally through the greater palatine canal in the extracranial pterygopalatine fossa, adjacent to the SPG. A transmitter coil was then placed on the patient's cheek to induce an electronic circuit.

**Table 5:** Characteristics of included human studies.

**References**	**Experimental subjects**	**Control group**	**Intervention**	**Protocol of stimulation**	**Ganglion target**	**Outcome of interest**	**Results**
Khurana et al. (60) (ImpACT-1)	Ischemic stroke patients (*n* = 98)	Historical control (*n* = 165)	Invasive electrical stimulation	Frequency: 10 Hz Intensity: 5 to 25 mA Pulse: 100–400 s Parameter: 3–4 h for 5–7 consecutive days	Sphenopalatine ganglion	mRS NIHSS	mRS: Patients treated with SPG stimulation had an average mRS lower by 0.76 than the historical controls(CMH test *p* = 0.001). NIHSS: The binary NIHSS success rate was 45%(38/84) in ImpACT-1 compared to 23.6%(39/165) in the NINDS controls (*p* = 0.0006). Functional outcomes were better in people treated with the Ischemic Stroke System
Bornstein 2019 (ImpACT-24A) (61)	Ischemic stroke patients (*n* = 202)	Sham stimulation (*n* = 101)	Invasive electrical stimulation	Frequency: 10 Hz Intensity: 5 to 25 mA Pulse: 100–400 s Parameter: 4-h session for 5 consecutive days	Sphenopalatine ganglion	Improved mRS score^*^; Substantial neurological recovery^**^ Functional independence^***^	(1) No statistical significance improve 3-month disability above expectations (2) Cortical involvement subtype showed statistical significance in improved mRS score, substantial neurological recovery but not functional independence
Bornstein 2019 (ImpACT-24B) (62)	Ischemic stroke patients (*n* = 555)	Sham stimulation (*n* = 519)	Invasive electrical stimulation	Frequency: 10 Hz Intensity: 5 to 25 mA Pulse: 100–400 s Parameter: 4-h session for 5 consecutive days	Sphenopalatine ganglion	Improved mRS score^*^; Functional independence^***^;	(1) No statistical significance improve 3-month disability above expectations (2) Cortical involvement subtype showed statistical significance in improved mRS score but not functional independence
Saver et al. (ImpACT-24M) (63)	Ischemic stroke patients (*n* = 50)	Historical control (*n* = 50)	Invasive electrical stimulation	Frequency: 10 Hz Intensity: 5 to 25 mA Pulse: 100–400 s Parameter: 4-h session for 5 consecutive days	Sphenopalatine ganglion	CBF NIHSS	CBF: Stimulation was associated with increase in CBF velocity and flow volume in the CCA during both peak systole and end-diastole with a 44% increase mean from baseline (*p* < 0.0001) NIHSS: The normalized change in NIHSS from day 1 to day 7 was significantly more favorable in the SPG stimulation than control patients. Evolution of the NIHSS in the SPG stimulation patients was from median 5 (IQR, 4–5) on day 1 to median 1 (IQR, 1–2) on day 7.
Sanchez et al. (52)	Healthy volunteers (*n* = 37)	N/A	Non-invasive magnetic stimulation	Frequency: 10 Hz Intensity: 1.0, 1.3, 1.6, 1.9T (0.8 and up) Pulse: 280 μs Parameter: Stimulation for 3 min after limit reached.	Geniculate ganglion	CBF	CBF: Clear responders to stimulation (i.e., a CBF increase of ≥ 25%) represents about a third of all volunteers.

* *Improved mRS score at 3 months beyond expectation*.

** *(NIHSS score ≤ 1 or improved ≥9) at 3 months*.

*** *(mRS 0–2) at 3 months*.

*CCA, common carotid artery; mRS, Modified Rankin scale; NIHSS, NIH Stroke scale; SPG, Sphenopalatine ganglion*.

Khurana et al. (60) reported the outcomes of the implant for augmentation of cerebral blood flow trial-1 (ImpACT-1). The prospective, single-arm, feasibility trial was conducted between 2006 and 2009. The trial recruited 98 subjects and reported no major safety concerns. At least one SAE was reported in 23 patients, with three classified as “related” or “possibly related” to the intervention, mostly due to the re-implantation and/or implant misplacement. Pain during stimulation (15/92) was the most frequently reported AE. Secondary efficacy analysis demonstrated a very slight reduction in mRS score (−0.76) between stimulation and historical controls. There was also a significant improvement in functional independence (0–2 mRS) at 90 days, with 48% of stimulated patients (40/84) reaching functional independence compared to 29% (48/165) in the historical control group (60).

Bornstein et al. (61) reported the outcomes of the implant for augmentation of cerebral blood flow trial-24A (ImpACT-24A), the follow-up efficacy trial of the ImpACT-1. The trial used a two-arm, randomized, double-blind design with a sham-control. Within the sham procedures the trans-oral device was implanted but no electrical current was activated. The trial was conducted from 2009 to 2011. The trial recruited patients with evidence of stroke in the anterior circulation, were able to undergo treatment within 24 h, and were ineligible for IV-tPA or endovascular thrombectomy. Bornstein et al. reported enrollment of 327 patients, however six exited prior to implantation and 18 had incomplete implantations. Patients were randomized 2:1, with 202 undergoing stimulation and 101 undergoing sham. Due to issues during implantation a new optic navigation system was introduced midway through the trial, however, only 75.7% of patients in the active group received stimulation. Due to the inconsistency of accurate device placement the trial was ended at the first interim analysis. A modified intent to treat (mITT) analysis looking at patients who underwent at least one full successful active or sham treatment found no benefits in either the primary outcome or any of the secondary endpoints. *Post-hoc* analysis demonstrated significant improvements within a subsection of the population that had confirmed cortical involvement (CCI), defined as patients with NIHSS score ≥10 and signs of hypodensity or tissue swelling in at least one cortical region on initial imaging (61).

The results of the follow-up trial, ImpACT24B, was conducted between 2011 and 2018 and reported by Bornstein et al. (62). Trial set-up was nearly identical to ImpACT24A, however, the CCI subsection was added as a primary outcome measure of interest. The trial protocol was changed several times, including the introduction of a new guidance system, neurostimulator, implantation technique, and electrical transmitter-control unit. Of the 1,078 patients recruited, 481 underwent stimulation and 519 underwent a sham operation. Again, no benefit in the mITT population was seen in any analysis, however the CCI subgroup showed statistically significant improvements in every endpoint. An inverted *U*-shape relationship between stimulation intensity and effectiveness in the CCI subgroup, with lower-mid-level stimulation indicated as most effective (62).

The ImpACT24M study, reported by Saver et al. (63), was a single-arm study assessing the effect of two changes to the SPG stimulation procedure required for implanting and optimizing the device. Primary outcomes were difference in 7-day NIH Stroke Scale (NIHSS), and proportion of patients with improvement in stroke symptoms, compared to historical controls (65). The 49 patients that received stimulation demonstrated a median NIHSS improvement of 75% compared to 50% in the historical control. Stimulation was also shown to significantly increase CBF and hand motor function within the cohort, but was not compared to the historical control (63).

There is one ongoing trial (NCT04014621) to determine if 6 h of SPG stimulation using the same procedure in the ImpACT studies will “freeze” the ischemic penumbra in patients with acute ischemic stroke and reduce brain tissue death. The primary outcome measure is volume of core expansion determined by CT scan post SPG stimulation. This trial is expected to complete in spring 2021.

### Non-invasive Ganglion Stimulation: Pre-clinical Studies

Six of the 26 publications identified assessed non-invasive interventions to stimulate facial nerves or ganglions (Table 4) (43–45, 51, 52, 56). Three studies used a non-invasive magnetic intervention, only one of which used an ischemic stroke model (43, 44, 56). Four studies used a non-invasive electrical intervention, all of which were tested in healthy models (43, 45, 51, 52). Borsody et al. (43) reported outcomes on both a magnetic and electrical non-invasive intervention, with each intervention targeting separate ganglions.

Magnetic stimulation interventions were primarily deployed through tesla coils located on either side of the subject's head. Magnetic intensities were reported in Tesla, and ranged from 0.5 to 1.9 T in the three studies (43, 44, 56). Non-invasive electrical stimulation was induced over 30–90 min, while the magnetic stimulation was induced over several 5 min increments with a 30 min recovery period.

All non-invasive stimulation studies found an increase in CBF compared to controls (43–45, 51, 52, 56), however, Borsody et al. (43) found a reduction in CBF following non-invasive electrical stimulation through the middle ear. They also reported the largest change in CBF following magnetic stimulation, with a 120% increase compared to baseline using a 10 Hz frequency at 1.5 T. Comparatively, Sanchez et al. (56) found the increase in CBF occurred throughout the brain without obvious preference for the hemisphere ipsilateral to stimulation, however this finding was not reported in any other study. No studies recorded neurological scores, and only two reported on infarct size (44, 45), with non-invasive stimulation significantly reducing infarct size compared to control in both studies.

### Non-invasive Ganglion Stimulation: Clinical Studies

Sanchez et al. (56) was the only clinical study that assessed the ability of non-invasive ganglion stimulation (Table 5). The geniculate ganglion was targeted and stimulated at 1, 1.3, 1.6, and 1.9 T in 280 μs pulses for 3 min in health volunteers. All tests were conducted using a single device (VitalFlow, NeuroSpring, California). CBF was found to significantly increase following stimulation, with higher intensity correlating to larger CBF flux. No adverse effects were reported within a 24-h follow-up.

## Discussion

There is a substantial amount of pre-clinical and clinical data to suggest that stimulation of the parasympathetic fibers of the facial nerve system is a promising option for rapid AIS treatment. Data from early trials with both invasive and non-invasive approaches have proven safe with very minimal reports of treatment-associated adverse events. Stimulation of the SPG or geniculate ganglion clearly results in increased CBF, and the technological means required to rapidly induce ganglion stimulation are certainly feasible. While the SPG has been the most well-studied ganglion in clinical trials to date, pre-clinical evidence suggests a variety of targets for the potential therapeutic development.

Despite the tremendous upside it poses for treating AIS, ganglion stimulation has yet to demonstrate clinically meaningful outcomes. To date the clinical trials for SPG stimulation via a trans-oral device have suffered from various methodological shortcomings that obscure the reasons behind the lack of positive results. A recurring issue with incorrect device placement resulted in the study protocols for both the ImpACT-24A and ImpACT-24B trials to be repeatedly modified during the trial course. The mid-trial change of the neurostimulator, implantation technique and electrical transmitter-control unit in the ImpACT-24B trial is especially disconcerting and was not addressed in the outcome analysis. Additionally, while the trials claim to be double or triple blinded, it is unclear how successful the blinding can be for the subject and physician when a key element of the intervention's deployment is mild facial discomfort. It is also not clear why the ImpACT-1 and ImpACT-24A were published almost 10 years after completion, and simultaneously with ImpACT-24B and ImpACT-24M.

Current strategies for employing ganglion stimulation to treat AIS suffer from three core limitations: (1) current trial design, (2), time-to-deployment, and (3) accessibility to trained physicians.

The ImpACT trials have focused on independently improving AIS-related disability, but their negative results suggest that tPA and mechanical thrombectomy will remain the gold standard for AIS treatment. It is therefore optimal for facial nerve stimulation to augment and improve the applicability of these existing treatments. There is a clear need for interventions that elongate the current window-of-efficacy (19), with extended door-to-treatment times leaving only 3–22% of AIS patients eligible for mechanical thrombectomy (66), and <5% eligible for tPA (67). Primary outcomes within ganglion stimulation trials should be focused on increasing the efficacy of mechanical thrombectomy for AIS patients that miss the current window-of-efficacy. The upcoming trial (NCT04014621) looking at penumbra “freezing” suggests future ImpACT studies involving invasive approaches may soon follow this suggestion.

However, if the goal of future trials is to stop the evolution of the penumbra prior to recanalization, it is key that ganglion stimulation protocols add minimal delay to the already extensive door-to-treatment time. The design of the current clinical stimulation devices and associated procedures are limited in their ability to accomplish this. The ImpACT-24B trial saw a 1.2-h difference in time from “last known well” on NIHSS to the first stimulation session between sham and control groups; this is likely due to the procedure associated with finding the ideal stimulation intensity, a step inherently missing from the sham procedure. Even without this optimization step, the implantation of the invasive device takes a reported average of 20 min, not including time for setting a sterile environment. This substantially eats away at thrombectomy's window-of-efficacy or the even shorter 60-min window for tPA induction (33, 68). Transfer time from intake hospitals to stroke centers is an ideal time for rapid ganglion stimulation, however, requirements associated with invasive implantation limit their applicability in an ambulatory care setting. Even the non-invasive methods used by Sanchez et al. (56) require TMS coils, which are often large and require substantial energy supply to induce magnetic fields.

The requirement of highly trained physicians, specialized imaging, and sterile fields further limit the currently studied innovations. Current invasive techniques for ganglion stimulation can be employed only by specialized physicians, demonstrated by 58% of implantations in the ImpACT-24B trial being performed by surgeons and anesthesiologists, with the remaining performed by neurologists (62). Implanting the device also requires an advanced optical guidance system that would likely only be available to stroke centers, limiting its ability to be implemented in peripheral hospitals prior to transfer.

The research to date on ganglion stimulation is extremely promising, but further innovation is required to find a workable integration of ganglion stimulation into current clinical procedures. An inherent focus must be on limiting the evolution of the penumbra and minimizing the size of the ischemic core, with the primary goal of elongating the window-of-efficacy for mechanical thrombectomy or tPA. Solutions should look to benefit these current treatment options instead of supplanting them, and find a way to be used primarily at peripheral non-stroke centers.

## Data Availability Statement

The original contributions presented in the study are included in the article/supplementary material, further inquiries can be directed to the corresponding author/s.

## Author Contributions

AC is the guarantor. TB conceptualized the study. TB and JR developed the search strategy and drafted the manuscript. JR extracted the data. All authors contributed to the development of the selection criteria, risk of bias assessment strategy, and data extraction criteria and also read, provided feedback, and approved the final manuscript.

## Conflict of Interest

The authors declare that the research was conducted in the absence of any commercial or financial relationships that could be construed as a potential conflict of interest.

## Publisher's Note

All claims expressed in this article are solely those of the authors and do not necessarily represent those of their affiliated organizations, or those of the publisher, the editors and the reviewers. Any product that may be evaluated in this article, or claim that may be made by its manufacturer, is not guaranteed or endorsed by the publisher.
